# Supplementary material for: Bioassay-Guided Isolation of New Flavonoid Glycosides from Platanus × acerifolia Leaves and Their Staphylococcus aureus Inhibitory Effects
Source: Molecules. 2022 Aug 23;27(17):5357. doi: 10.3390/molecules27175357 (PMC9457999; doi:10.3390/molecules27175357)
Supplement: Supplementary file 1 [file molecules-27-05357-s001.zip › molecules-1847742-supplementary.pdf]

Supporting Information

Bioassay Guided Isolation of New Flavonoid Glycosides from *Platanus* ×*acerifolia* Leaves and Their *Staphylococcus aureus* Inhibitory Effects

Xi-Ying Wu [1,3,†], Yu Tang [4,†,*], Ezzat E. A. Osman [1,5,†], Jiang Wan [2], Wei Jiang [6], Guo-Xun Yang [1], Juan Xiong [1], Quangang Zhu [3,*], and Jin-Feng Hu [1,2,*]

[1] Department of Natural Medicine, School of Pharmacy, Fudan University, Shanghai 201203, China

[2] Institute of Natural Medicine and Health Products, School of Pharmaceutical Sciences, Zhejiang Provincial Key Laboratory of Plant Ecology and Conservation, Taizhou University, Zhejiang 318000, China

[3] Shanghai Skin Disease Hospital, Tongji University School of Medicine, Shanghai 200443, China

[4] College of Pharmaceutical Sciences, Zhejiang University, Hangzhou 310058, China

[5] Department of Medicinal Chemistry, Theodor Bilharz Research Institute, Kornaish El-Nile St., Giza 12411, Egypt

[6] School of Life Science and Technology, Wuhan Polytechnic University, Hubei 430023, China

* Correspondence: jfhu@fudan.edu.cn (J.-F.H.); zhuqg@shskin.com (Z.G.); yutang@zju.edu.cn (Y.T.); Tel.: +86-21- 51980172 (J.-F.H)

[†] These authors contributed equally to this work.

Contents

Figure S1. ^{1}H NMR spectrum of compound **1** (CD_3OD, 400 MHz).

Figure S2. ^{13}C NMR spectrum of compound **1** (CD_3OD, 600 MHz).

Figure S3. HSQC spectrum of compound **1** (CD_3OD, 600 MHz).

Figure S4. ^{1}H-^{1}H COSY spectrum of compound **1** (CD_3OD, 600 MHz).

Figure S5. HMBC spectrum of compound **1** (CD_3OD, 600 MHz).

Figure S6. DEPT 135 spectrum of compound **1** (CD_3OD, 600 MHz).

Figure S7. HRESIMS data of compound **1**

Figure S8. ^{1}H NMR spectrum of compound **2** (CD_3OD, 400 MHz).

Figure S9. ^{13}C NMR spectrum of compound **2** (CD_3OD, 600 MHz).

Figure S10. HSQC spectrum of compound **2** (CD_3OD, 600 MHz).

Figure S11. ^{1}H-^{1}H COSY spectrum of compound **2** (CD_3OD, 600 MHz).

Figure S12. HMBC spectrum of compound **2** (CD_3OD, 600 MHz).

Figure S13. HRESIMS data of compound **2**.

Figure S1. ^{1}H NMR spectrum of compound **1** (CD_3OD, 400 MHz).

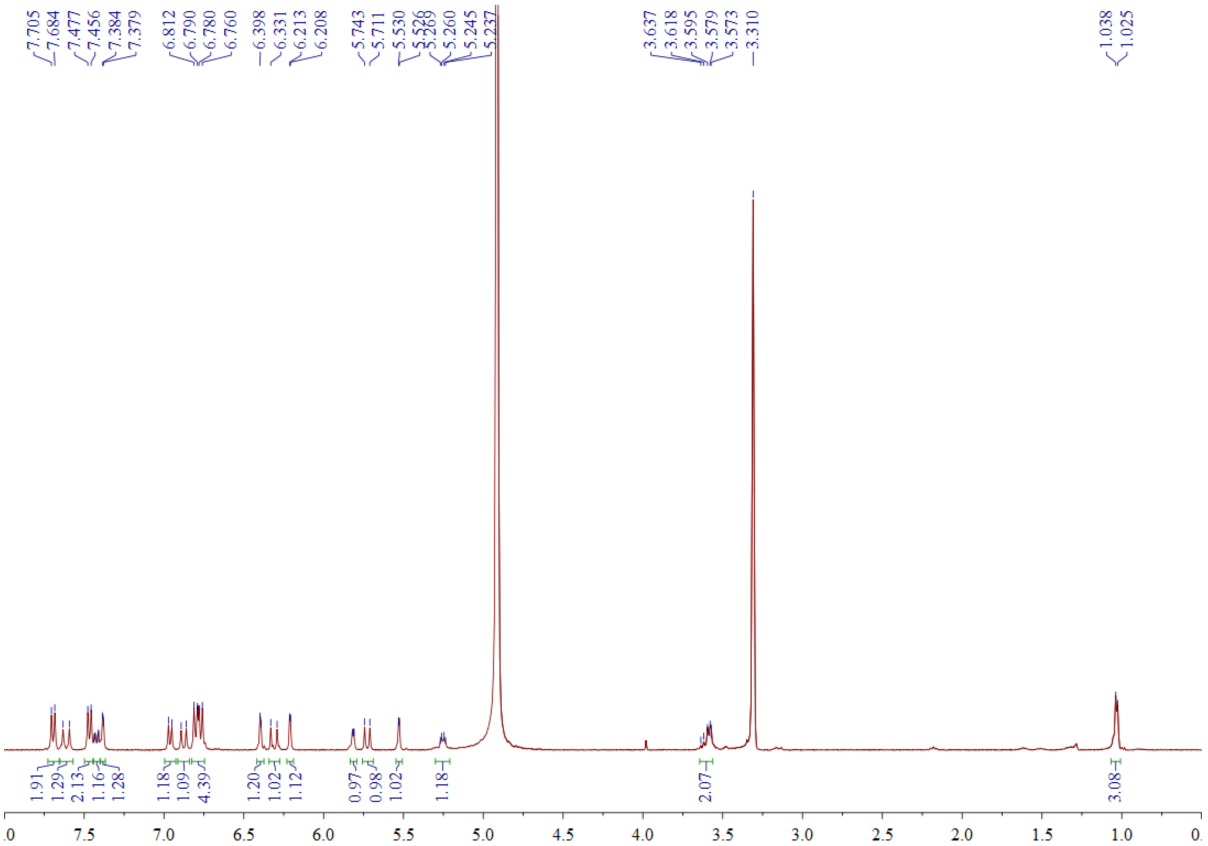

Figure S2. ^{13}C NMR spectrum of compound **1** (CD_3OD, 600 MHz).

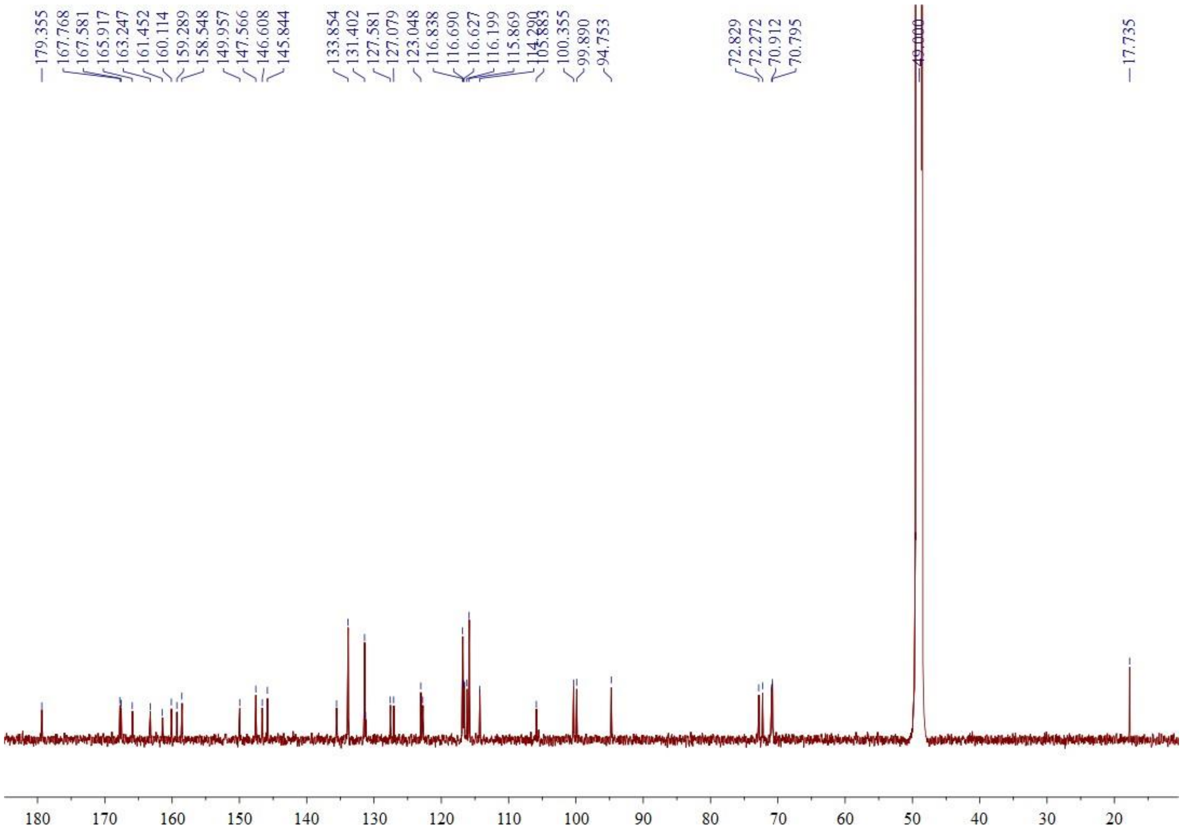

Figure S3. HSQC spectrum of compound **1** (CD_3OD, 600 MHz).

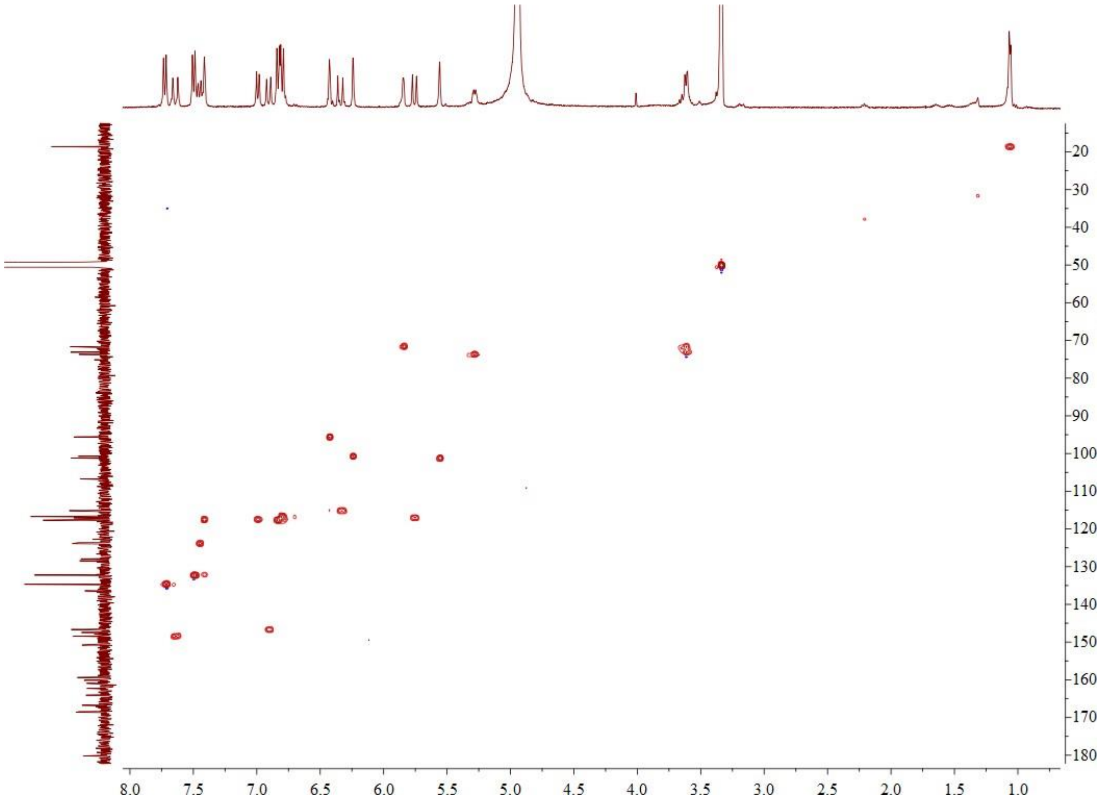

Figure S4. ^1H-^1H COSY spectrum of compound **1** (CD_3OD, 600 MHz).

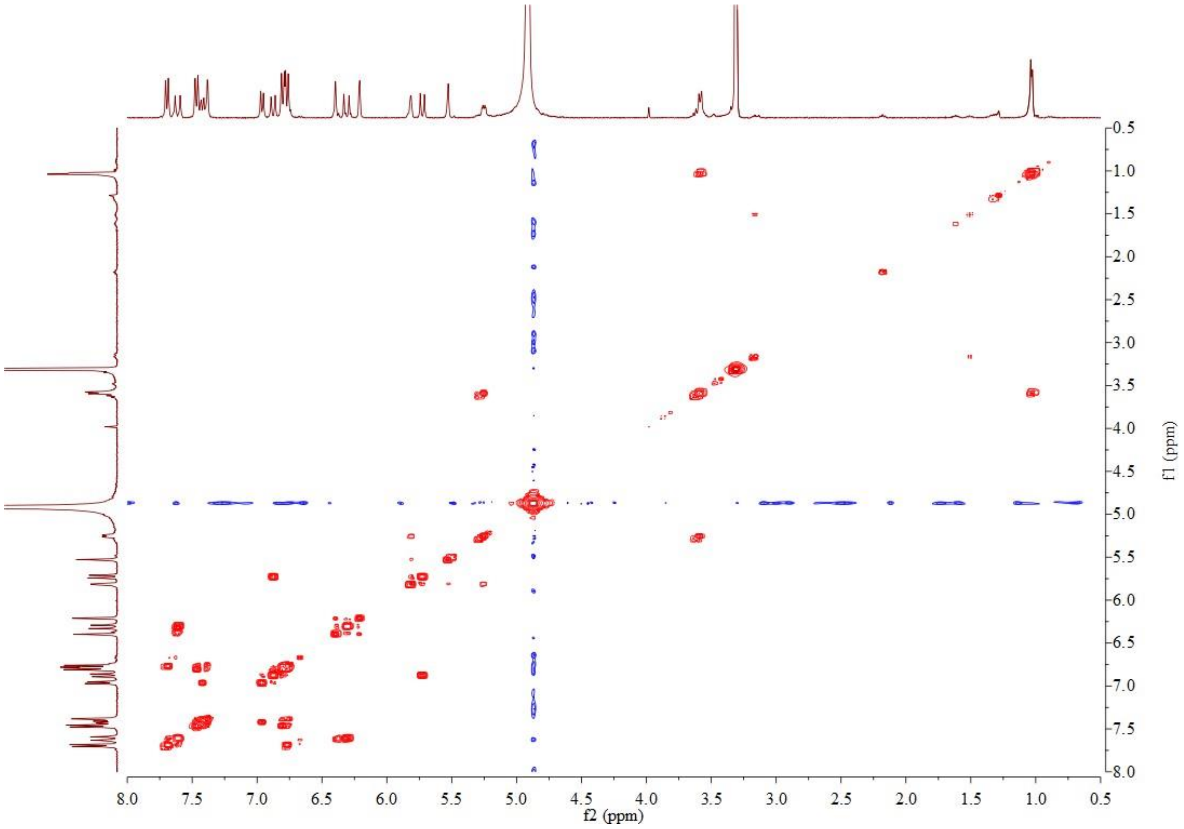

Figure S5. HMBC spectrum of compound **1** (CD_3OD, 600 MHz).

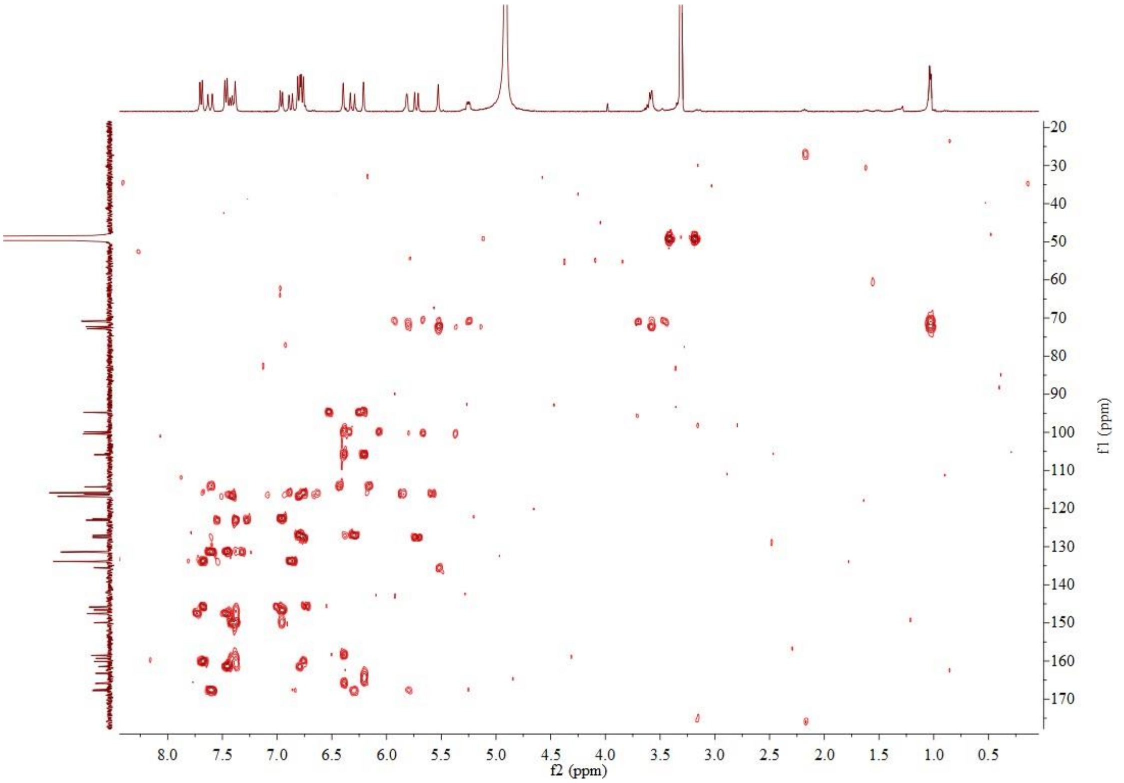

Figure S6. DEPT 135 spectrum of compound **1** (CD_3OD, 600 MHz).

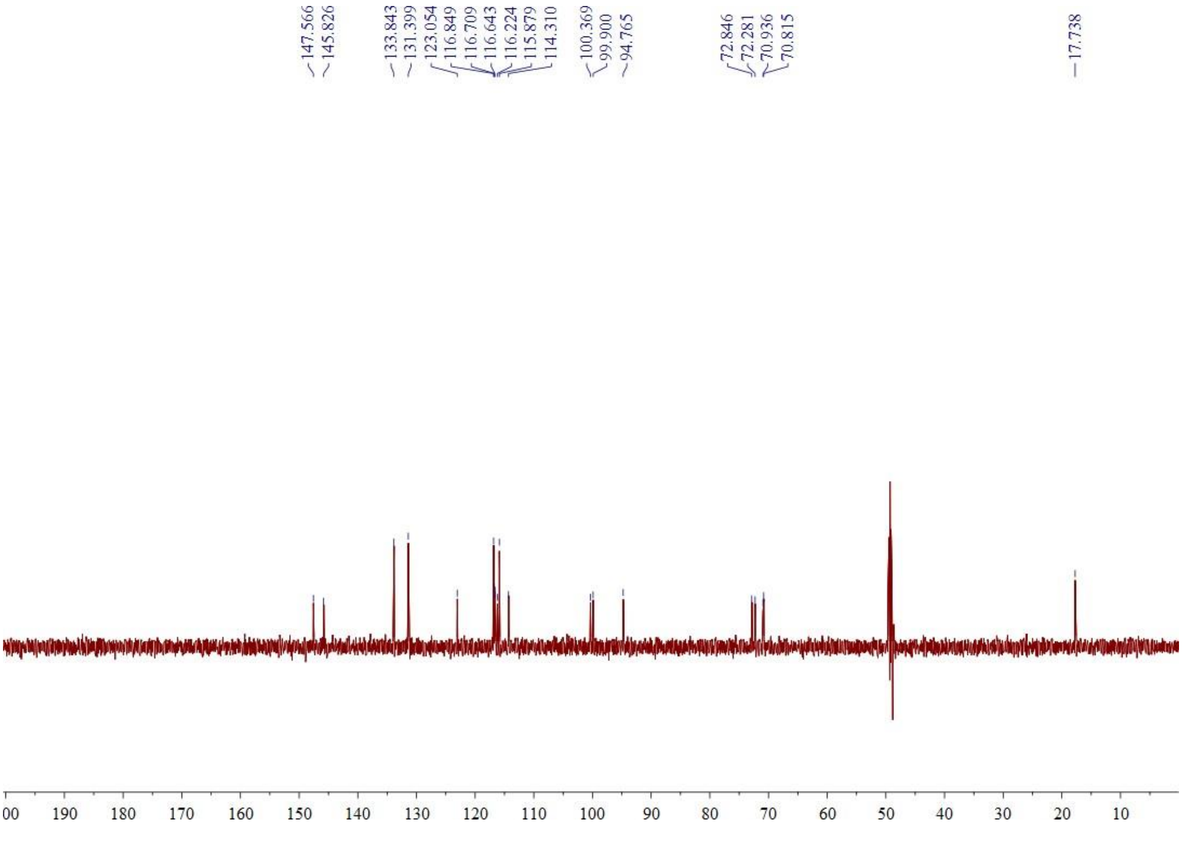

Figure S7. HRESIMS data of compound **1**

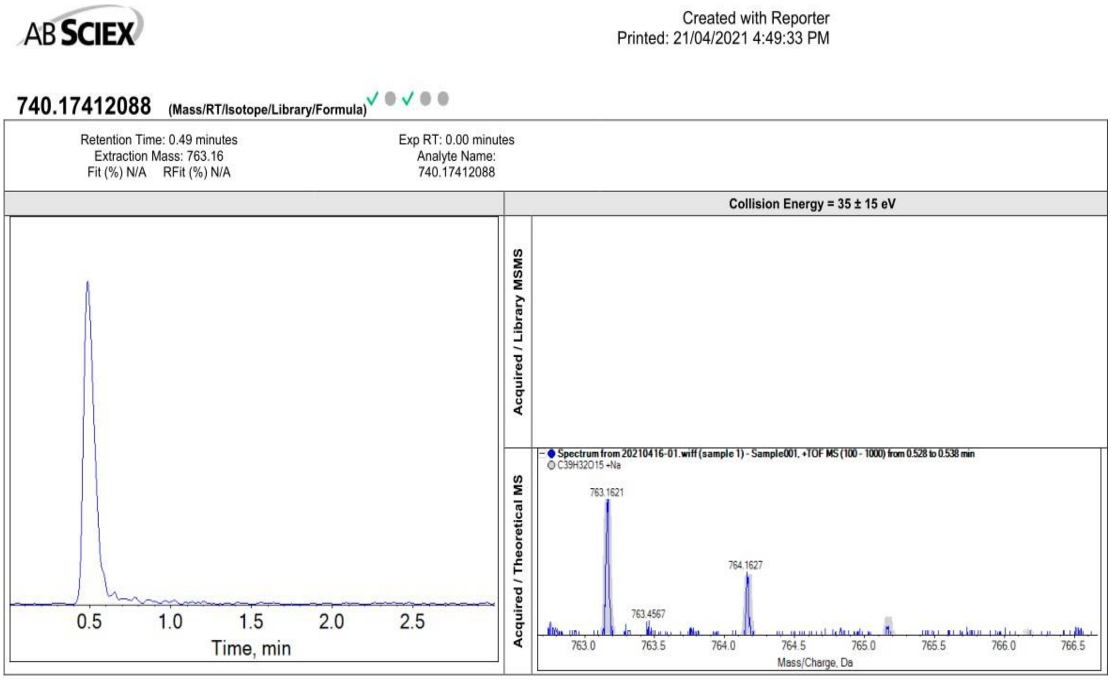

	Compound Name (Library Hit)	Score	Formula	Intensity	Threshold	Expected m/z	Found at m/z	Error (ppm)	Expected RT (min)	Found RT (min)	RT Delta (min)	Isotope Diff (%)	Library Score (%)
	740.17412088	88%	C39H32O15	6747	5	763.1633	763.1621	-1.6	0.00	0.49	0.49	6.6%	N/A

Figure S8. ^{1}H NMR spectrum of compound **2** (CD_3OD, 400 MHz).

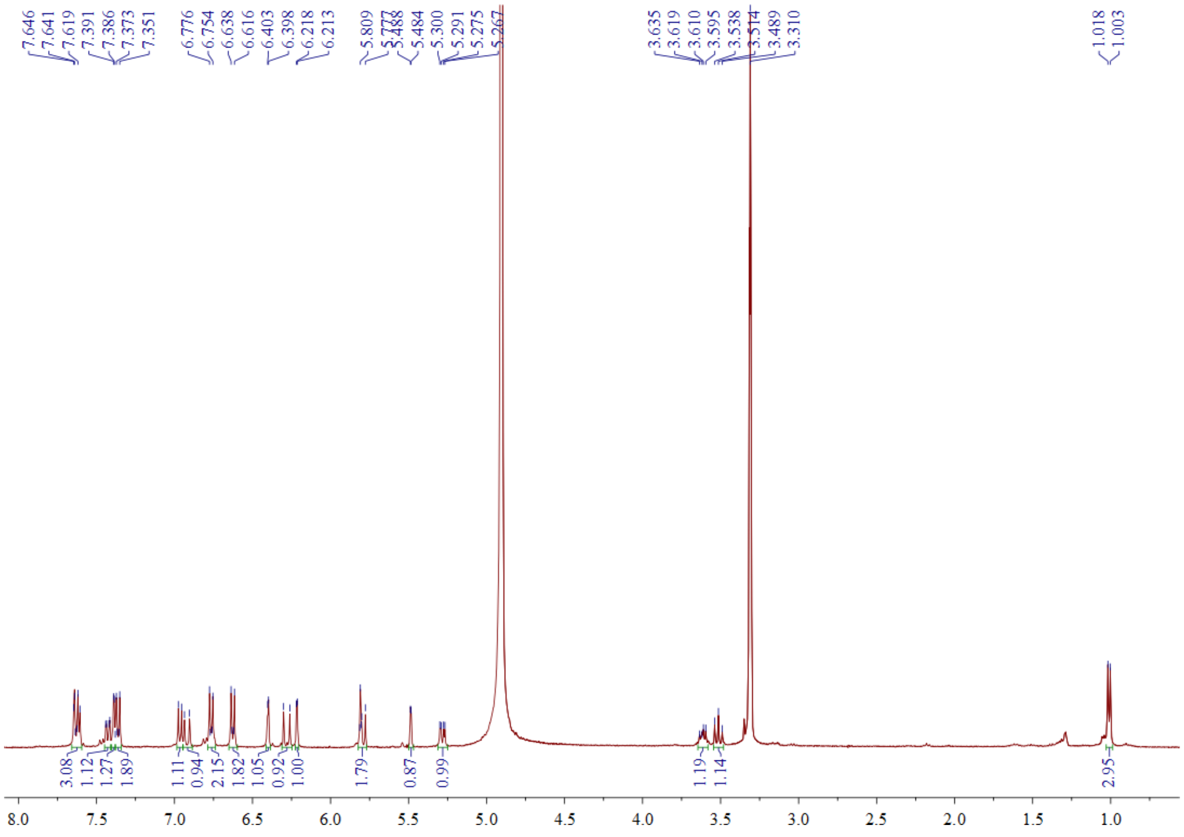

Figure S9. ^{13}C NMR spectrum of compound **2** (CD_3OD, 600 MHz).

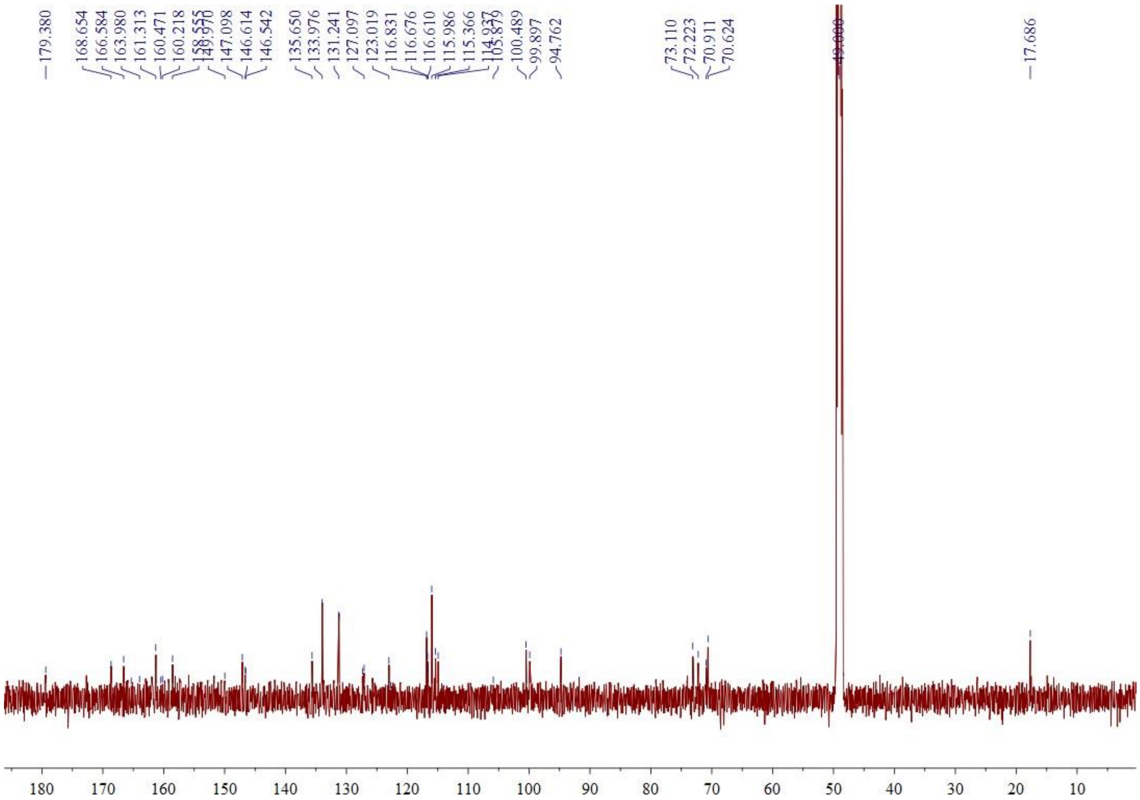

Figure S10. HSQC spectrum of compound **2** (CD_3OD, 600 MHz).

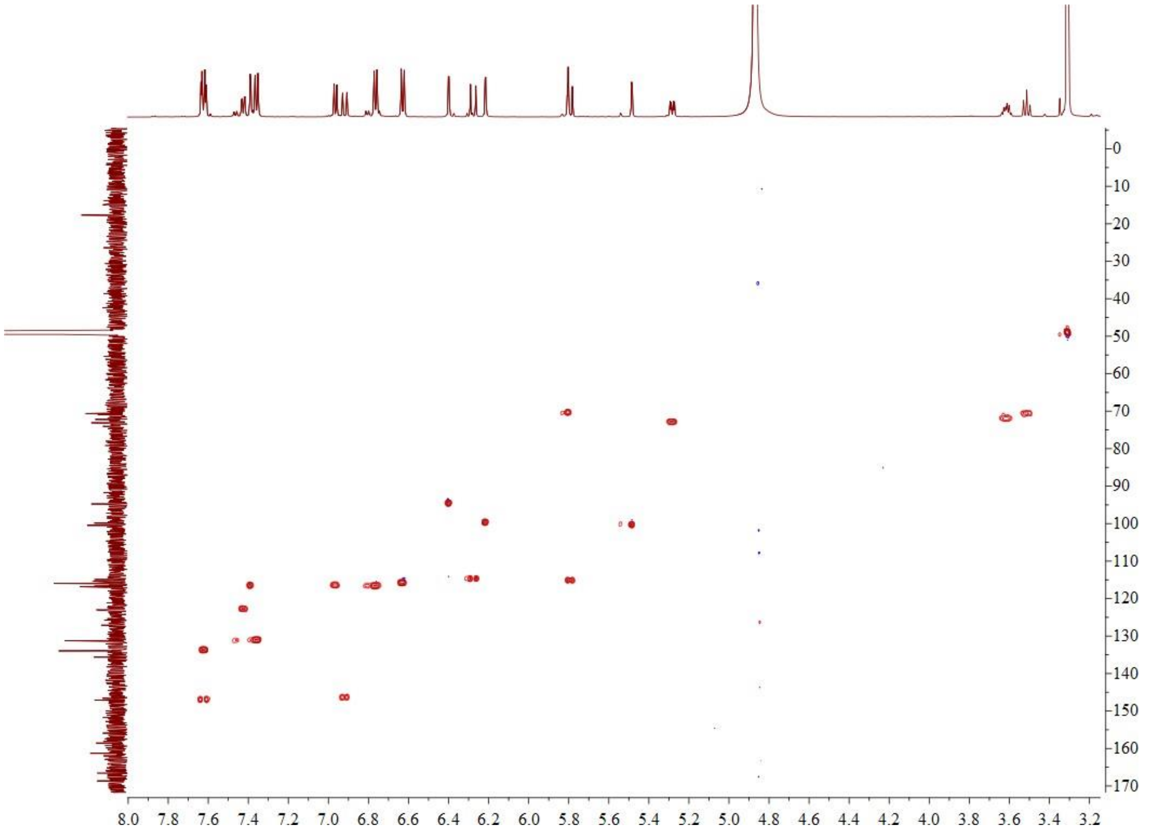

Figure S11. ^{1}H-^{1}H COSY spectrum of compound **2** (CD_3OD, 600 MHz).

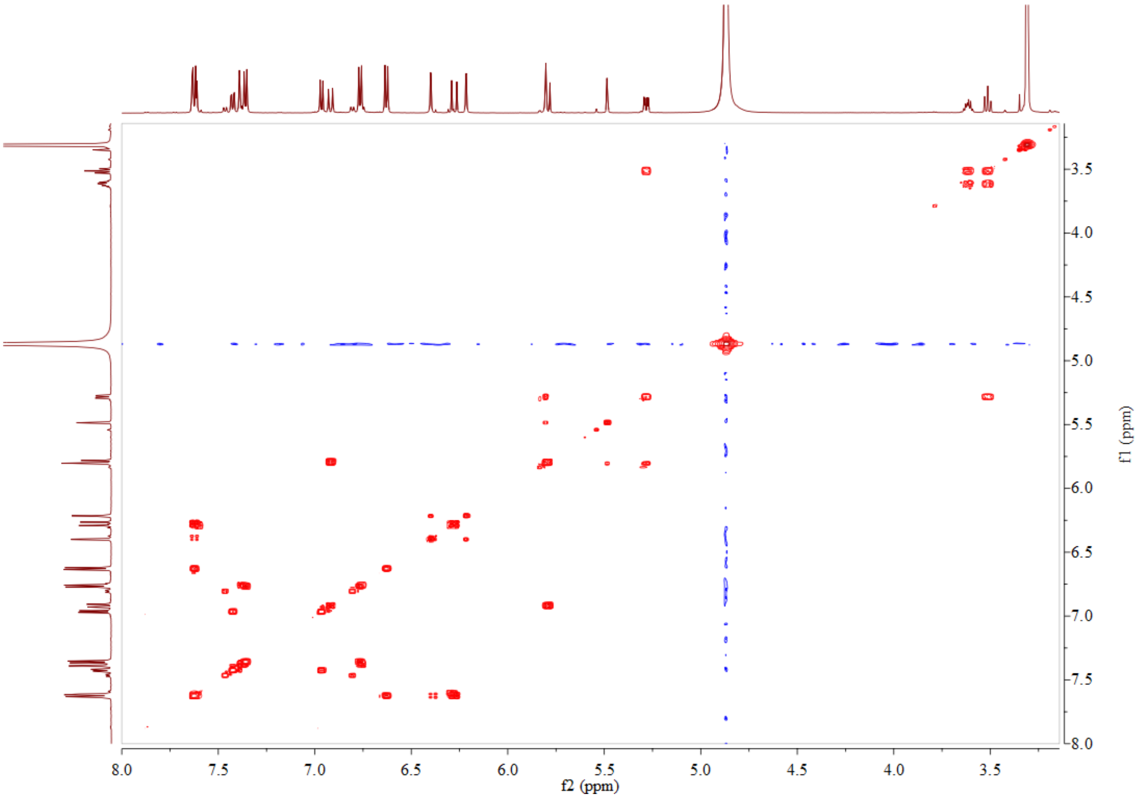

Figure S12. HMBC spectrum of compound **2** (CD_3OD, 600 MHz).

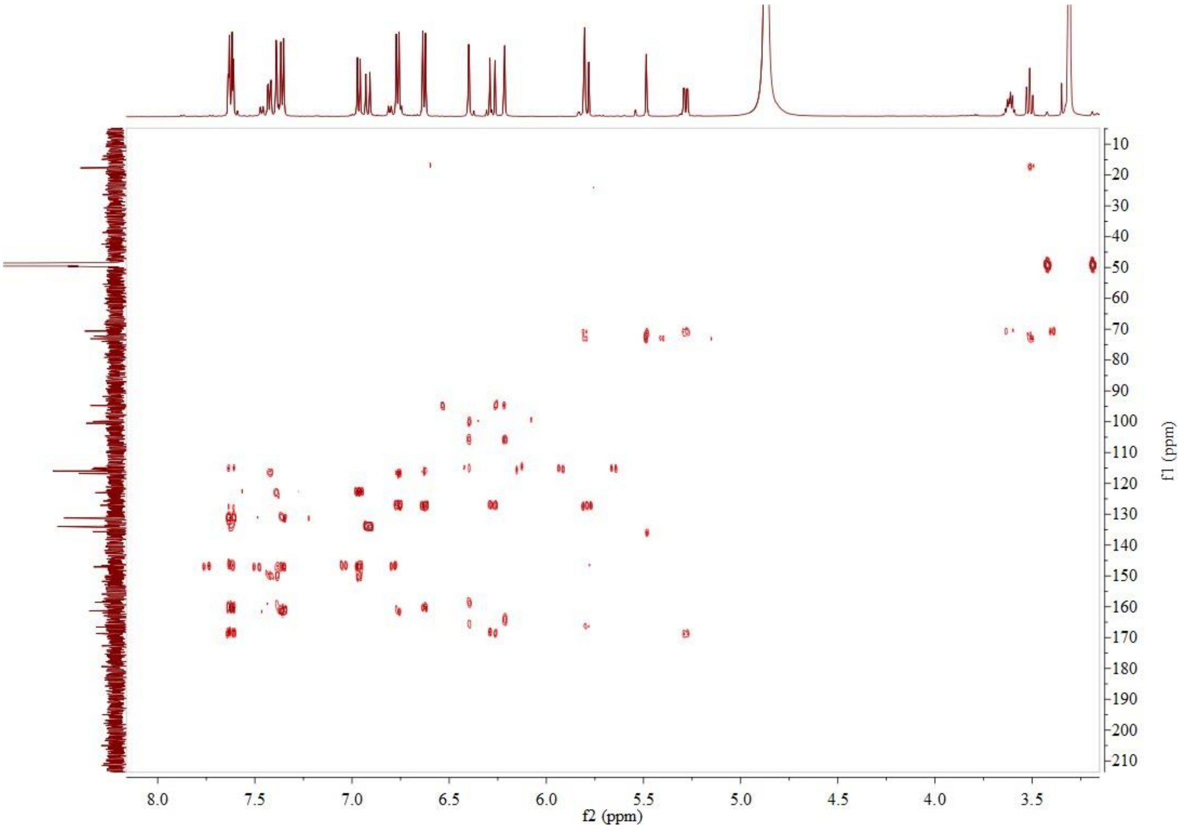

Figure S13. HRESIMS data of compound **2**.

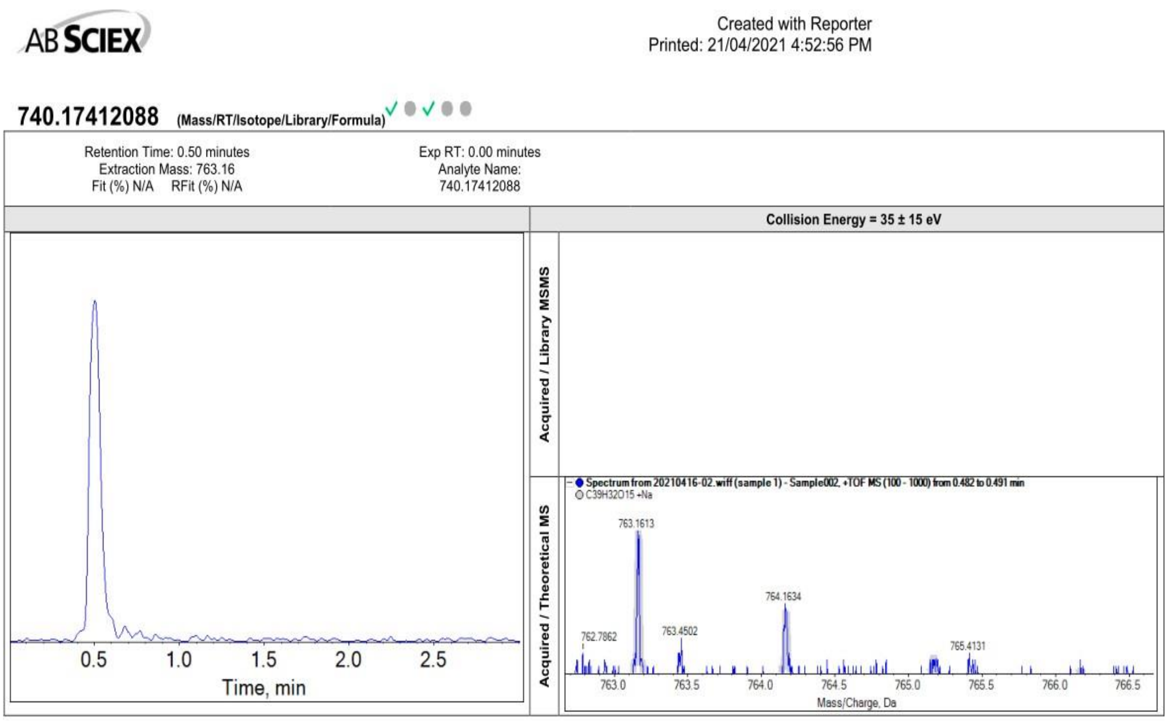

Mass/RT/Isotope/Library/Formula	Compound Name (Library Hit)	Score	Formula	Intensity	Threshold	Expected m/z	Found at m/z	Error (ppm)	Expected RT (min)	Found RT (min)	RT Delta (min)	Isotope Diff (%)	Library Score (%)
✓ ● ✓ ● ●	740.17412088	78%	C39H32O15	2747	5	763.1633	763.1613	-2.7	0.00	0.50	0.50	12.6%	N/A
